# Supplementary material for: Single-Molecule Methods to Investigate Mechanisms of Transcription by RNA Polymerase of Mycobacterium tuberculosis
Source: bioRxiv. 2026 Apr 14:2026.03.27.714832. Originally published 2026 Mar 28. Preprint. [Version 2] doi: 10.64898/2026.03.27.714832 (PMC13041941; doi:10.64898/2026.03.27.714832)
Supplement: 1 [file NIHPP2026.03.27.714832v2-supplement-1.pdf]

bioRxiv preprint doi: https://doi.org/10.64898/2026.03.27.714832; this version posted April 14, 2026. The copyright holder for this preprint (which was not certified by peer review) is the author/funder, who has granted bioRxiv a license to display the preprint in perpetuity. It is made available under aCC-BY 4.0 International license.

Supplementary material

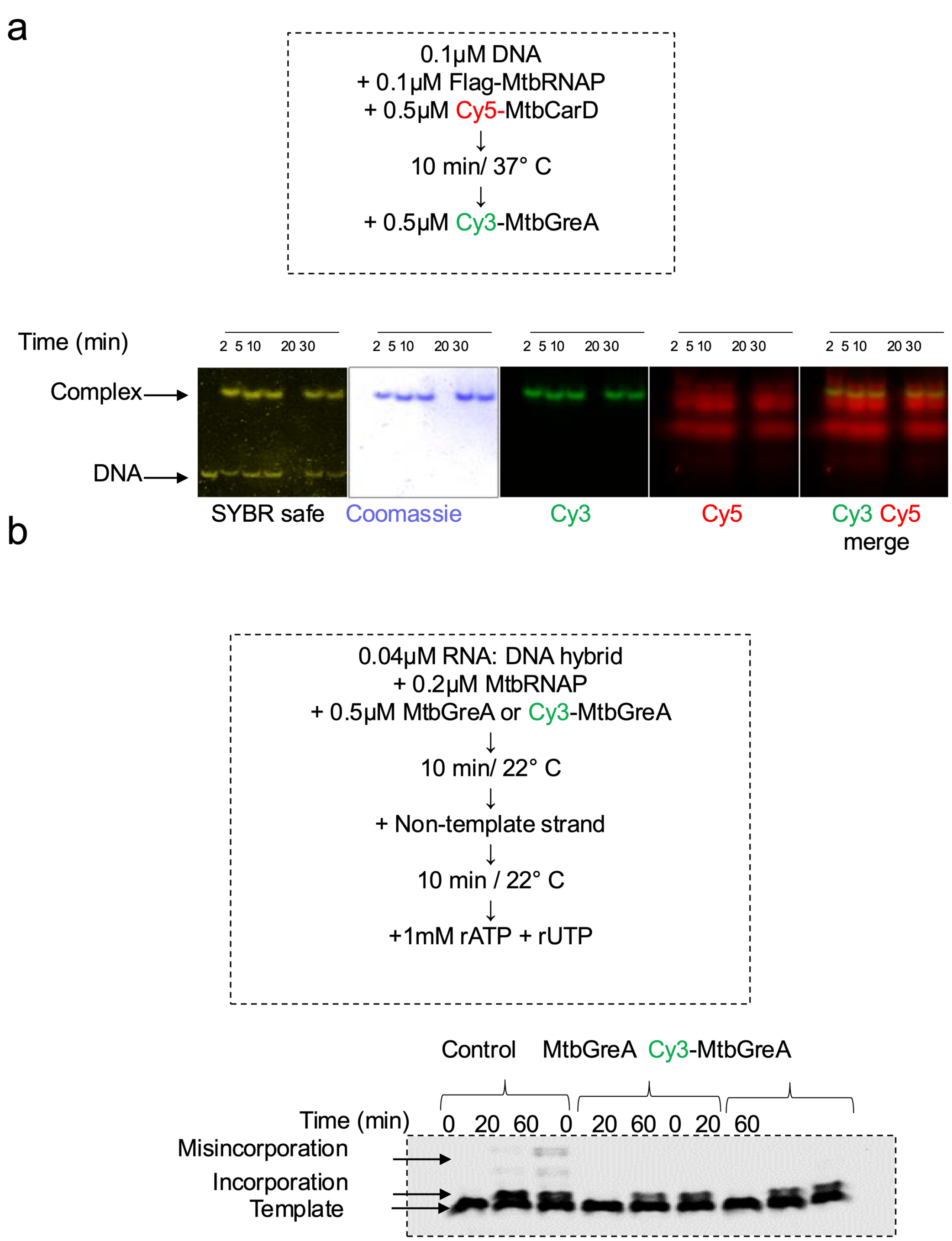

Fig. S1. Evaluation of Cy5-MtbCarD and Cy3-MtbGreA functional activities

a. An electrophoretic mobility shift assay to verify a transcription initiation complex formation with Cy5-MtbCarD.
b. A misincorporation bulk assay to verify Cy3-MtbGreA activity.

bioRxiv preprint doi: https://doi.org/10.64898/2026.03.27.714832; this version posted April 14, 2026. The copyright holder for this preprint (which was not certified by peer review) is the author/funder, who has granted bioRxiv a license to display the preprint in perpetuity. It is made available under aCC-BY 4.0 International license.

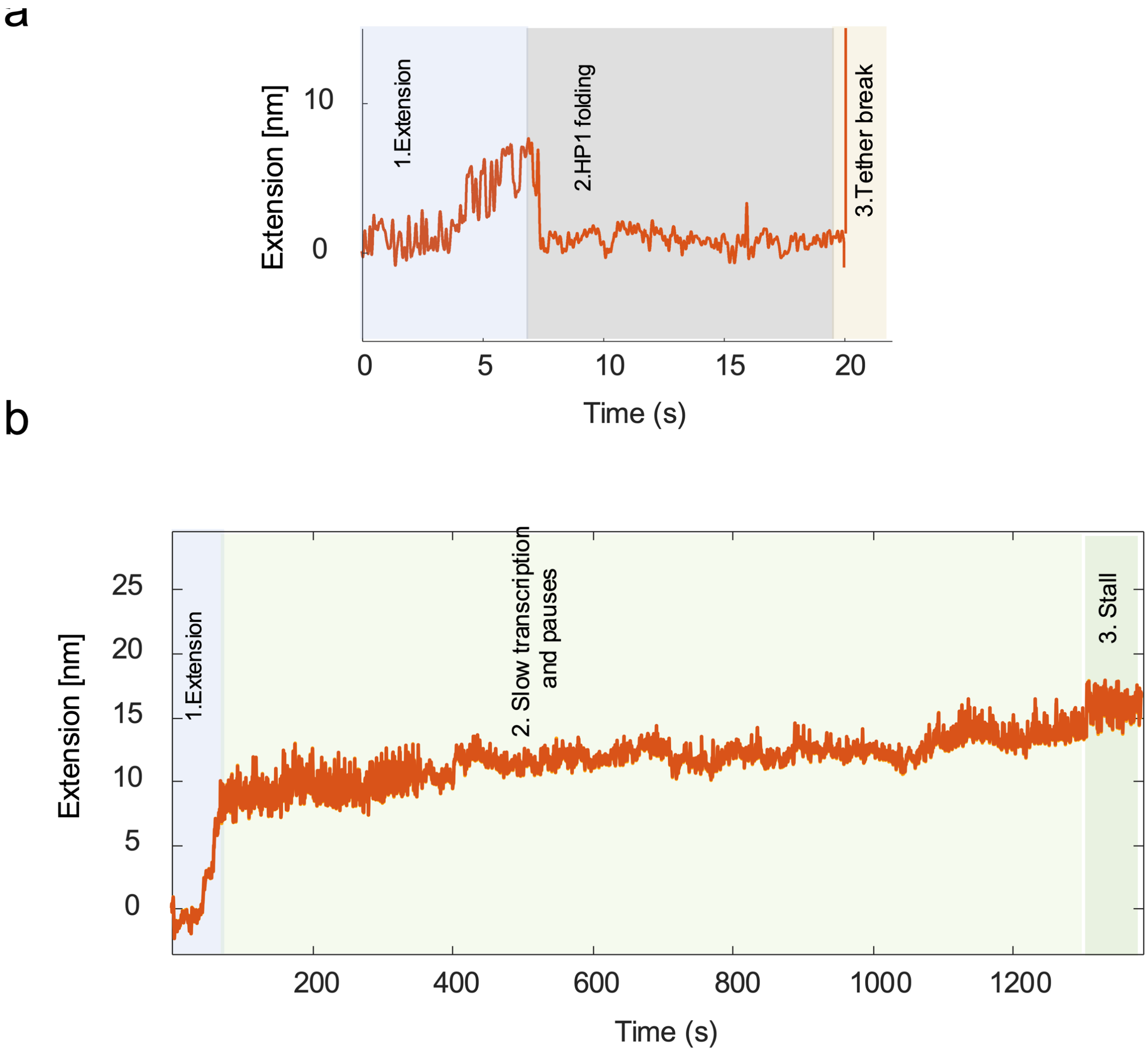

Fig. S2. Other RNA co-folding trajectories
a. One shortened trajectory.
b. One trajectory indicated that MtbRNAP paused, slowly transcribed, and finally stalled.

bioRxiv preprint doi: https://doi.org/10.64898/2026.03.27.714832; this version posted April 14, 2026. The copyright holder for this preprint (which was not certified by peer review) is the author/funder, who has granted bioRxiv a license to display the preprint in perpetuity. It is made available under aCC-BY 4.0 International license.

SI. Table 1

Name	Sequence
~ 290 bp DNA template	Promoter AC50, transcription start site (TSS), C-less cassette, and a fragment of rpoB gene in *M. tuberculosis* H37Rv.<br><br>GCCAAGCTTGCATGCCGACGGCCAGTGAATTCAAATATTTGTTGTTAACTCTTGACAAAAGTGTT<br>AAAAGCGGCTAGTATTTAAAGGGATGGATGACCATCTCTCTAGACCCACCTGGGTTGGTGTGCC<br>CACAGCGGCTGGAAGGTCGACGCCGCCAAGGGGGTTCCGGACTGGGCCGCCAGGCTGCCCGA<br>CGAACTGCTCGAGGCGCAGCCGAACGCCATTGTGTCGACGCCGGTGTTCGACGGCGCCCAGGA<br>GGCCGAGCTGCAGGGCCTGTTGTCGTGCACGCTGCCCAACCGC
~ 2.8 kb DNA template (total length ~ 2.9 Kb)	Promoter AC50, transcription start site (TSS), C-less cassette, and fragments of rpoB and rpoC genes in M. tuberculosis H37Rv, including the intergenic region.<br><br>TTGCATGCCGACGGCCAGTGAATTCAAATATTTGTTGTTAACTCTTGACAAAAGTGTTAAAAGC<br>GGCTAGTATTTAAAGGGATGGATGACCATCTCTCTAGACCCACCTGGGTTGGTGTGCCCACAGC<br>GGCTGGAAGGTCGACGCCGCCAAGGGGGTTCCGGACTGGGCCGCCAGGCTGCCCGACGAACT<br>GCTCGAGGCGCAGCCGAACGCCATTGTGTCGACGCCGGTGTTCGACGGCGCCCAGGAGGCCG<br>AGCTGCAGGGCCTGTTGTCGTGCACGCTGCCCAACCGCGACGGTGACGTGCTGGTCGACGCCG<br>ACGGCAAGGCCATGCTCTTCGACGGGCGCAGCGGCGAGCCGTTCCCGTACCCGGTCACGGTTG<br>GCTACATGTACATCATGAAGCTGCACCACCTGGTGGACGACAAGATCCACGCCCGCTCCACCGG<br>GCCGTACTCGATGATCACCCAGCAGCCGCTGGGCGGTAAGGCGCAGTTCGGTGGCCAGCGGTT<br>CGGGGAGATGGAGTGCTGGGCCATGCAGGCCTACGGTGCTGCCTACACCCTGCAGGAGCTGTT<br>GACCATCAAGTCCGATGACACCGTCGGCCGCGTCAAGGTGTACGAGGCGATCGTCAAGGGTGA<br>GAACATCCCGGAGCCGGGCATCCCCGAGTCGTTCAAGGTGCTGCTCAAAGAACTGCAGTCGCT<br>GTGCCTCAACGTCGAGGTGCTATCGAGTGACGGTGCGGCGATCGAACTGCGCGAAGGTGAGG<br>ACGAGGACCTGGAGCGGGCCGCGGCCAACCTGGGAATCAATCTGTCCCGCAACGAATCCGCAA<br>GTGTCGAGGATCTTGCGTAAAGCTGTCGCAAAATTACTAAACCCGTTAGGGGAAAGGGAGTTA<br>CGTGCTCGACGTCAACTTCTTCGATGAACTCCGCATCGGTCTTGCTACCGCGGAGGACATCAGG<br>CAATGGTCCTATGGCGAGGTCAAAAAGCCGGAGACGATCAACTACCGCACGCTTAAGCCGGAG<br>AAGGACGGCCTGTTCTGCGAGAAGATCTTCGGGCCGACTCGCGACTGGGAATGCTACTGCGGC<br>AAGTACAAGCGGGTGCGCTTCAAGGGCATCATCTGCGAGCGCTGCGGCGTCGAGGTGACCCGC<br>GCCAAGGTGCGTCGTGAGCGGATGGGCCACATCGAGCTTGCCGCGCCCGTCACCCACATCTGG<br>TACTTCAAGGGTGTGCCCTCGCGGCTGGGGTATCTGCTGGACCTGGCCCCGAAGGACCTGGAG<br>AAGATCATCTACTTCGCTGCCTACGTGATCACCTCGGTCGACGAGGAGATGCGCCACAATGAGC<br>TCTCCACGCTCGAGGCCGAAATGGCGGTGGAGCGCAAGGCCGTCGAAGACCAGCGCGACGGC<br>GAACTAGAGGCCCGGGCGCAAAAGCTGGAGGCCGACCTGGCCGAGCTGGAGGCCGAGGGCG<br>CCAAGGCCGATGCGCGGCGCAAGGTTCGCGACGGCGGCGAGCGCGAGATGCGCCAGATCCGT<br>GACCGCGCGCAGCGTGAGCTGGACCGGTTGGAGGACATCTGGAGCACTTTCACCAAGCTGGCG<br>CCCAAGCAGCTGATCGTCGACGAAAACCTCTACCGCGAACTCGTCGACCGCTACGGCGAGTACT<br>TCACCGGTGCCATGGGCGCGGAGTCGATCCAGAAGCTGATCGAGAACTTCGACATCGACGCCG<br>AAGCCGAGTCGCTGCGGGATGTCATCCGAAACGGCAAGGGGCAGAAGAAGCTTCGCGCCCTC<br>AAGCGGCTGAAGGTGGTTGCGGCGTTCCAACAGTCGGGCAACTCGCCGATGGGCATGGTGCTC<br>GACGCCGTCCCGGTGATCCCGCCGGAGCTGCGCCCGATGGTGCAGCTCGACGGCGGCCGGTTC<br>GCCACGTCCGACTTGAACGACCTGTACCGCAGGGTGATCAACCGCAACAACCGGCTGAAAAGG<br>CTGATCGATCTGGGTGCGCCGGAAATCATCGTCAACAACGAGAAGCGGATGCTGCAGGAATCC<br>GTGGACGCGCTGTTCGACAATGGCCGCCGCGGCCGGCCCGTCACCGGGCCGGGCAACCGTCC<br>GCTCAAGTCGCTTTCCGATCTGCTCAAGGGCAAGCAGGGCCGGTTCCGGCAGAACCTGCTCGG<br>CAAGCGTGTCGACTACTCGGGCCGGTCGGTCATCGTGGTCGGCCCGCAGCTCAAGCTGCACCA<br>GTGCGGTCTGCCCAAGCTGATGGCGCTGGAGCTGTTCAAGCCGTTCGTGATGAAGCGGCTGGT<br>GGACCTCAACCATGCGCAGAACATCAAGAGCGCCAAGCGCATGGTGGAGCGCCAGCGCCCCCA<br>AGTGTGGGATGTGCTCGAAGAGGTCATCGCCGAGCACCCGGTGTTGCTGAACCGCGCACCCAC

bioRxiv preprint doi: https://doi.org/10.64898/2026.03.27.714832; this version posted April 14, 2026. The copyright holder for this preprint (which was not certified by peer review) is the author/funder, who has granted bioRxiv a license to display the preprint in perpetuity. It is made available under aCC-BY 4.0 International license.

<table>
<tr><td></td><td>CCTGCACCGGTTGGGTATCCAGGCCTTCGAGCCAATGCTGGTGGAAGGCAAGGCCATTCAGCT
GCACCCGTTGGTGTGTGAGGCGTTCAATGCCGACTTCGACGGTGACCAGATGGCCGTGCACCT
GCCTTTGAGCGCCGAAGCGCAGGCCGAGGCTCGCATTTTGATGTTGTCCTCCAACAACATCCTG
TCGCCGGCATCTGGGCGTCCGTTGGCCATGCCGCGGCTGGACATGGTGACCGGGCTGTACTAC
CTGACCACCGAGGTCCCCGGGGACACCGGCGAATACCAGCCGGCCAGCGGGGATCACCCGGA
GACTGGTGTCTACTCTTCGCCGGCCGAAGCGATCATGGCGGCCGACCGCGGTGTCTTGAGCGT
GCGGGCCAAGATCAAGGTGCGGCTGACCCAGCTGCGGCCGCCGGTCGAGATCGAGGCCGAGC
TATTCGGCCACAGCGGCTGGCAGCCGGGCGATGCGTGGAACCGCGAGACCGGATCCCCGGGT
ACCGAGCTCGAATTCA - digoxygenin</td></tr>
<tr><td>~ 890 bp DNA template</td><td>Promoter A1T7, transcription start site for dinucleotide rApU, G-U-less Cassette, Terminator t_{tuf} from M. tuberculosis H37Rv with the U-track, with the alternative termination sites in bold (the arrows showing the long hairpin stem and short hairpin stem), and the previous termination region. The underlined text corresponds to the DNA sequence that codes for the 5' of the nascent RNA that is designed to be hybridized with the 5' single strand overhand of one of the DNA handles to form a single molecule tether.

GGCCGCCAGATCTTCCGGATGGCTCGAGTTTTTCAGCAAGATCAGTCACGACGTTGTAAAACGACGGCCAG
TGAATTCGGGCCCTTATCAAAAAGAGTATTGACTTAAAGTCTAACCTATAGGATACTTACAGCCATCCCACC
ACAACCACCACAACCACCACAACAACAACCACAACAACTGAAGGATCCCGGGCCCGTCGACTGCAGAGGC
CTGCATGCGACGAAGGTCTGCGTTTCGCGATCCGCGAGGGTGGCCGCACCGTGGGCGCCGGCCGGGTCA
CCAAGATCATCAAGTAGGT
--→ ←--
CTACCGGCCACCAGACGCAAAAGAACATGATGGGCGCACCAGCGCCCATCATGTTCTTTTGCGTCTGCTC
--------------→ ←---------------
GCGAAAATGCCCAGCGTCATGGATTCTAGCAACTAACTTACCTGTTGAAATTCGAACACCCAAACAACTTGT
TAATATCTATTCGAAGCGAATGCAGATTGAAGAAACCTTCCGAGACTTGAAAAGTCCTGCCTACGGACTAG
GCCTACGCCATAGCCGAACGAGCAGCTCAGAGCGTTTTGATATCATGCTGCTAATCGCCCTGATGCTTCAA
CTAACATGTTGGCTTGCGGGCGTTCATGCTCAGAAACAAGGTTGGGACAAGCACTTCCAGGCTAACACAGT
CAGAAATCGAAACGTACTCTCAACAGTTCGCTTAGGCATGGAAGTTTTGCGGCATTCTGGCTACACAATAA
CAAGGGAAGACTTACTCGTGGCTGCAACCCTACTAGCTCAAAATTTATTCACACATGGTTACGCTTTGGGG
AAATTATGAGGGGATCTCTCAGAGCTAAATATTCAATATGTTCCTCTTGACCAACTTTATTCTGCATTTTTTT
TGAACGAGGTTTAGAGCA</td></tr>
</table>

SI. Table 2

Step	Expected Length (nm)
1. Transcribe linker 1 + Half of HP1	9.6
2. Fold HP1, just the linker remains	6.5
3. Transcribe Linker 2 + Half of SS2	11.8
4. Fold SS2, just Linker 1 + Linker 2 remains	8.8
5. Transcribe Linker 3 + Half of t_{tuf}	29.5
6. Fold t_{tuf}, just Linker 1+2+3 remains	20.4
